# Obesity as disruptor of the female fertility

**DOI:** 10.1186/s12958-018-0336-z

**Published:** 2018-03-09

**Authors:** Erica Silvestris, Giovanni de Pergola, Raffaele Rosania, Giuseppe Loverro

**Affiliations:** 1 Interdisciplinary Department of Medicine, Section of Obstetrics and Gynecology, University of Bari Aldo Moro, P.za G.Cesare, 11-70124 Bari, Italy; 2 Departmentof Biomedical Sciences and Human Oncology, Section of Internal Medicine and Clinical Oncology, University of Bari Aldo Moro, P.za G.Cesare, 11-70124 Bari, Italy

**Keywords:** Obesity, Infertility, Adipokines, Anovulation, Oocytes

## Abstract

Both obesity and overweight are increasing worldwide and have detrimental influences on several human body functions including the reproductive health. In particular, obese women undergo perturbations of the ‘hypothalamic pituitary ovarian axis’, and frequently suffer of menstrual dysfunction leading to anovulation and infertility. Besides the hormone disorders and subfertility that are common in the polycystic ovary syndrome (PCOS), in obesity the adipocytes act as endocrine organ. The adipose tissue indeed, releases a number of bioactive molecules, namely adipokines, that variably interact with multiple molecular pathways of insulin resistance, inflammation, hypertension, cardiovascular risk, coagulation, and oocyte differentiation and maturation. Moreover, endometrial implantation and other reproductive functions are affected in obese women with complications including delayed conceptions, increased miscarriage rate, reduced outcomes in assisted conception treatments.

On the contrary, weight loss programs through lifestyle modification in obese women, have been proven to restore menstrual cyclicity and ovulation and improve the likelihood of conception.

## Background

Major functions of the female gonads include the production of gametes, the oocytes, and sex hormones that control the development of the female secondary sexual characters and support the pregnancy. In physiology, these functions are cyclically exerted between puberty and menopause, and are regulated by endocrine and paracrine factors that interact with several cell types located in ovaries [1]. Thus, besides other pathological outcomes, dysfunctional or altered regulation of these mechanisms can either directly or indirectly result in infertility.

According to the American Society of Reproductive Medicine Practice Committee [2], infertility is a disease generally defined as *failure to conceive after twelve or more months of attempts of natural fertilization* and is a rising problem in our society today. The WHO worldwide estimation suggests that this pathology currently affects up to 50–80 millions of women, with a variable incidence that in several instances may raise up to about 50% of all women [3].

A number of factors appear to concur to provoke infertility in women mainly including ovulatory dysfunction, tubal, cervical and/or uterine factors, as well as endometriosis although 20–30% of cases remain unexplained [4]. Recently, the effects of lifestyle on female reproductive health have received great attention as well as the body mass index (BMI), foods and nutrients, sport and physical activity, stressing jobs and other conditions, are currently widely claimed as having a negative impact on the female fertility [5].

Here, we critically review the potential influence of the altered metabolic state on the reproductive health of women, focusing the effects of obesity on fertility, and the management of infertility in obese and overweight women.

### Pathophysiology of the woman infertility

A number of differential conditions concur to affect the woman fertility. Several of them are strictly related to the pathophysiology of reproductive organs whereas others are mainly dependent on occupational aspects including the lifestyle factors as the hypercaloric nutrition usually resulting in metabolic derangements and increase in the body weight promoting obesity.

Below are listed the major causes of infertility belonging to both of these pathogenic conditions as next detailed.

#### Dysfunction of reproductive organs

- ***Deregulated ovarian function*** – Since the ovulation is the result of a complex balance and interaction of hormones, any alteration of these mechanisms may influence its physiology. The most common cause of ovulation failure includes the polycystic ovary syndrome (PCOS) [6]. This condition is related to the arrest of the follicle maturation resulting in formation of small follicles, defective ovulation and dysmenorrhea. Other causes include malfunctions of the hypothalamus or pituitary gland leading to the production of immature eggs, or the result of invasive surgery for repeated ovarian cysts as well as ovulatory disorders dependent on extraovarian pathologies including either hypothyroidism or hyperthyroidism.
- ***Tubal infections*** *–* The factors responsible for tubal disease are different and derive from post-infection damage, obstruction, pelvic adhesion even after surgery, and probably undefined others. In this contest, the pelvic inflammatory disease is a major etiologic event of anatomic and functional disorders of tubae and is predominantly associated to infections by *Chlamydia Trachomatis* and *Neisseria Gonorrhoea* which can ultimately lead to tubal related infertility [7].
- ***Endometriosis*** – It is a chronic condition characterized by the growth of endometrial tissue in areas other than the uterine cavity, most commonly in the pelvic cavity, including the ovaries. Tubal damage can occur as a result of the chronic inflammation associated to the growing endometrioid tissue and approximately 20–30% of women with endometriosis suffer of subfertility [8].
- ***Cervical factor*** *–* The cervix plays an important role in the reproduction activity. It provides the passage way for sperms, allowing them to access into the uterine cavity and ultimately into the fallopian tubes. The ability of the sperm to gain access to the upper tract is influenced by the cervical mucus within the cervical canal. Therefore, all conditions modifying the mucosal film of the cervix may concur to prevent the progression of sperms toward the tubae.
- ***Uterine factors*** *–* Dysfunction in uterus such as defect of adhesion molecules, polyps, submucous fibroids, asymptomatic tumors, recurrent miscarriages as well as other endometrial pathologies and infections, may dramatically affect the blastocyst engraftment. Exploration of the uterine cavity by hysteroscopy is thus mandatory in the presence of unexplained bleeding.
- ***Unknown causes of infertility*** *–* In addition to the described major reproductive pathologies, other causes including hormone alterations, constitutive defects of inflammatory soluble factors and/or chronic alterations of metabolic pathways of reproductive organs, are related to infertility and remain to be assessed in clinical investigative studies [9].

#### Lifestyle and infertility

Recently, great interest has been focused on several lifestyle factors including the emotional state, typical of stress or anxiety condition, modifiable habits and ways of life that can greatly influence overall health and well being. Below are listed several of those factors for which there is a defined evidence of a negative impact on fertility.

- ***Psychological stress for social life and work*** – Particularly in western countries there is a larger incidence of depression and anxiety, which is partially related to the lifestyle and to a number of stressing jobs. Thus, the symptoms related to both depression and anxiety have been reported as more prominent in infertile than in fertile women. In a series of studies it has been indeed described that infertile women are more likely to experience higher levels of psychological distress [10], as well as high levels of reactive depression [11], and increased likelihood of anxiety [12]. A probability based study also suggested that the general distress levels are lower in the presence of higher socioeconomic status, or conversely, higher in the presence of highly stressing jobs, poor economic conditions or missing work. These aspects concur to generate a persistent condition of psychological stress in addition to the detrimental well being feeling, may alter the physiologic maturation of oocytes [13].
- ***Smoking*** *–* Although in the absence of defined molecular mechanisms, there is an apparently negative influence of smoking resulting in reduced fertility in female smokers. Several effects on negative reproductive consequences of chronic smoking include rapid depletion of ovarian follicles, conception delay, increased risk of spontaneous miscarriage in both natural and assisted conception cycles as well as increased risk of birth defects [14].
- ***Drugs, caffeine and alcohol*** *–* There are a number of daily or occasionally assumed drugs that are known to impact fertility. Non-steroidal anti-inflammatory drugs, commonly used to treat pain or inflammation are defined ovulation inhibitors whereas cytotoxic chemotherapy drugs used in anticancer treatments are primarily responsible of the ovarian failure in women [15]. On the other hand, higher introduction of caffeine has been associated with an increased risk of pregnancy loss whereas in alcohol abusers the liver metabolic alterations and/or psyconeurologic damages concur with stress factors to restrain the oocyte maturation.
- ***Diet and variations of body weight*** – Inadequate diets as those with poor calories and protein content or, vice versa, based on habitual hypercaloric food assumption, leading to severe thinness or overweight, definitely affect the ovarian function and increase the risk of women infertility. To this regard, it has been reported that the time to conceive is longer in women with BMI over 25 kg/m^2^ or, vice versa, less than 19 kg/m^2^, and that obesity and overweight are significantly associated with decreased pregnancy rates, increased requirements for gonadotrophins and higher miscarriage events. High BMI is also associated with adverse pregnancy outcomes such as gestational diabetes, hypertension and premature lebor.

Evidence on the effect of diet composition in fertility is scarce. Several studies investigating the effect of various dietary factors on fertility have been completed on data from extended studies including 116,678 women in the Nurses’ Health Study II. In these reports, it has been described a reduced risk of fertility due to ovulatory disorder in women whose diet included prevalently foods with low glycaemic content and limited intake of nutrients. This dietary restriction, indeed, may increase insulin resistance, such as lower trans fatty acid intake [16] thus supporting the pivotal role exerted by the glucose homeostasis and insulin sensitivity in the normal ovulatory function and fertility as occurs in women with normal weight.

- ***Physical activity*** *–* The excess in physical activity could be closely linked to considerable negative effects on the whole body including skeletal pathologies or functional derangements in endocrine, cardiovascular, reproductive and central nervous systems. In contrast with the vantages of sport activities in decreasing the abdominal fat, blood glucose, lipids and insulin resistance as well as in regulating the menstrual cyclicity, ovulation and fertility due to the lowered testosterone levels and increase of sex hormone binding globulin (SHBG), the intensively exerted agonistic sports may typically produce the ‘*female athlete triad’*, a syndrome characterized by amenorrhea, osteoporosis and eating disorder as defined by the American College of Sports Medicine [17].
- ***Environmental pollutants*** – Based on several investigative studies, the OSHA (Occupational Safety and Health Administration, USA) provided evidence that chronic exposure to specific chemicals as organic solvents, aromatic amines, heavy metals, phytodrugs, vegetal toxins and others is associated with reduced fertility as well as to increased tendency to either occasional or recurrent abortions [18]. The pathogenic mechanisms leading to unsuccessful fertility by these conditions are unclear and further studies are required to clarify the effect of environmental pollutants on female fertility.

The growing interest and the amount of research in this field emphasize the role of lifestyle factors in affecting the woman fertility and particularly obesity may represent a major condition in which both alteration of metabolic pathways and inflammatory factors concur to reduce the oocyte viability leading to subfertility or infertility.

### Molecular and endocrinological influence of obesity on woman fertility

In female mammals, a number of evolutionary mechanisms are enrolled to integrate environmental, nutritional and hormonal cues in order to guarantee reproduction in normal energetic conditions and, vice versa, to inhibit it in case of food scarcity. This metabolic strategy could be an advantage in nutritionally poor environments, but nowadays is affecting women’s health since the energy states may influence the female reproductive health in conditions of under or overweight.

Obesity and strenuous physical activity are conditions that alter the profiles of specific hormones such as insulin and adipokines and, thus, definitely impair the women fertility. Furthermore, there is undoubtedly a tight interconnection between energy metabolism and fertility, above all in females.

Obesity is a common problem among women of reproductive age. Obesity and overweight involves an abnormal and excessive fat accumulation that negatively affects the health status. According to the World Health Organization (WHO), if BMI is equal or greater than 25 kg/m^2^, it is considered overweight, whereas the BMI higher than 30 kg/m^2^ defines obesity [19]. The WHO reports that 60% of women are overweight in the United States and in most European countries while 30% of the female population are obese. Moreover, 6% of all women are morbidly obese (BMI ≥ 35 kg/m^2^) [20]. The negative effects of obesity on the reproductive physiology are known, since obese women frequently undergo menstrual irregularity with ovulatory disorders, endometrial pathology, and infertility.

The impact of obesity on reproductive function, especially ovulatory disorders, are mainly attributable to endocrine mechanisms [21], which interfere with neuroendocrine and ovarian functions, and reduce the ovulation omeostatic [22]. In obese women, gonadotropin secretion is affected as effect of the increased peripheral aromatization of androgens to estrogens while the insulin resistance and hyperinsulinemia lead to hyperandrogenemia. Furthermore, the sex hormone-binding globulin (SHBG), growth hormone (GH), and insulin-like growth factor binding proteins (IGFBP) are decreased and leptin levels are increased. Thus, the neuroregulation of the hypothalamic-pituitary-ovarian (HPO) axis may be severely deranged while the obese condition also increases the risk of miscarriage, poor pregnancy outcomes, and impaired fetal well being [23].

The risk of infertility has been shown to be threefold higher in obese than in non-obese women, and several studies have demonstrated that the obese females need longer time to pregnancy. In particular, two studies performed in large cohorts of Danish women planning pregnancies showed an inverse relation of fecundity with respect to the BMI increase [24]. It is noteworthy that obese women remain subfertile even in the absence of ovulatory dysfunction and Gesink and coworkers showed reduced fecundity in eumenorrheic obese women by examining a large American cohort of more than 7000 women in whom the probability of spontaneous conception linearly declined with each BMI point > 29 kg/m^2^ as in a parallel study completed in a Dutch cohort of more than 3000 women with normal cycles [25].

Moreover, fertility in obese women seems to be impaired also in assisted conception programs. In fact, overweight and obesity are also associated with negative outcomes for patients undergoing in vitro fertilization (IVF) due to the poor oocyte quality, as well as the lower preimplantation rate and uterine receptivity. Therefore, in these women it is strongly recommend the weight loss in order to improve the fertility functions [26].

The molecular and endocrinological aspects of obesity, its effects and consequences on the reproductive system as well as benefits from modifying lifestyles improving the reproductive outcomes, are next reviewed.

#### Adipokines as major fat tissue soluble products

The adipose tissue is considered an endocrine organ that plays important roles in the regulation of many physiological events, such as reproduction, immune response, glucose and lipid metabolism, through the secretion of a variety of bioactive cytokines, named adipokines, commonly involved in metabolic regulation and inflammatory processes.

During the last years, the dysfunction of the adipose tissue has been implicated in the pathophysiology of infertility based on recently discovered effects of adipokines. Their normal levels are critical to maintain the integrity of hypothalamus-pituitary-gonadal axis as well as to regulate ovulatory processes, successful embryo implantation, and in general the physiologic pregnancy.

The family of adipokines includes the adipose-specific cytokines, namely secreted by adipocytes, such as leptin, adiponectin (APN), resistin, visfatin and omentin, and the non adipose specific cytokines such as retinol binding protein-4 (RBP4), lipocalin-2 (LCN2), chemerin, interleukin 6 (IL6), interleukin 1β (IL1β), and tumor necrosis factor α (TNFα). The panel of adipokines is depicted in Table 1.

**Table 1:** Major effects of adipokines in fertility of the obese woman

Adipokines	Serum Levels	Effects
Leptin	↑	Inhibits insulin induced ovarian steroidogenesis Inibiths LH stimulated E2 production by the granulosa cells
Adiponectin	↓	Plasma insulin levels increase
Resistin	↑	Causes insulin resistance
Visfatin	↑	Increased insulin sensitivity
Omentin	↓	Increased insulin sensitivity
Chemerin	↑	Negatively regulates FSH_induced follicular steroidogenesis

*LH* Luteinizing hormone, *FSH* Follicle stimulating Hormone, *E2* Estradiol

Abnormal levels of these factors have been shown to be strongly associated with both insulin resistance (IR) and type 2 diabetes mellitus (T2DM) and in patients with the PCOS, a severe dysfunction of adipose tissue has been observed leading to over-production of pro-inflammatory adipokines such as TNFα and, concurrently, reduction of a few “beneficial adipokines” such as APN. Peculiarities of each bioactive cytokine are next summarized.

**Fig. 1:**
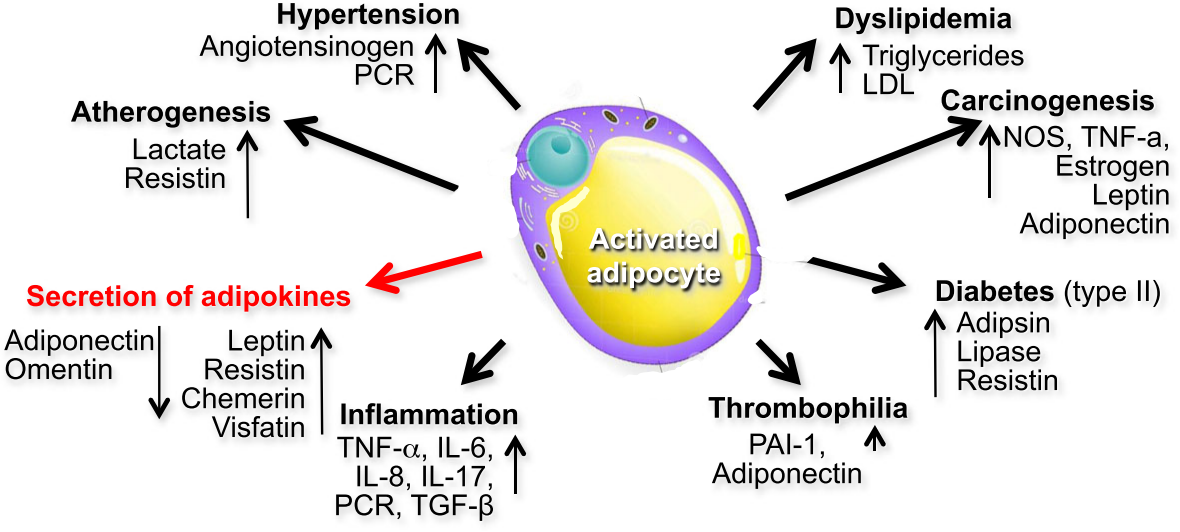
Functional properties of adipocites

- ***Leptin*** *-* Leptin is a 167-amino acid protein encoded by the “*ob”* gene. It is an important hormone involved in the regulation of food intake, energy balance, and body weight. Leptin is the first discovered adipokine to realize the endocrine functions of adipose tissue Fig. 1. It’s predominantly secreted by adipose tissue and largely available in many organs as stomach, placenta, hypothalamus, pituitary, and mammary gland. The leptin receptor (LEPR) is a single transmembrane domain receptor that is highly expressed in the choroid plexus. There are six isoforms of the LEPR (Ob-Ra, b, c, d, e, and f) due to the alternative RNA splicing. Leptin is constitutively secreted by adipocytes in relation to the extent of the adipose mass and is abundant in subjects with extensive abdominal fat. It suppresses the food intake and promotes energy expenditure mainly via its direct effects on hypothalamic neurons, and is thus considered an anti-obesity hormone. Its levels decrease with fasting and increase after food intake [27].

Higher circulating levels of leptin may lead to chronic down-regulation of LEPR in the brain of obese women, and higher leptin-BMI ratios may well explain lower rates of pregnancy with IVF. Jain et al., found that the amplitude of LH pulsatility was significantly lower in eumenorrheic obese women, thus suggesting a central defect. Moreover, higher serum levels of leptin in obese women correlate with higher levels of leptin in the follicular fluid. In vitro studies have shown that leptin affects steroidogenic pathways in granulosa cells and decreases both estrogen and progesterone production in a dose-dependent manner [28]. This negative effect of obesity on the oocyte physiology could have downstream effects on endometrial receptivity and embryo implantation.

- ***Adiponectin*** *-* Adiponectin (APN) is the most abundant secreted protein expressed exclusively in adipose tissue. There are three forms of APN: at *low molecular weight* (APN-LMW); at *medium molecular weight* (APN-MMW); and at *high molecular weight* (APN-HMW). Three receptors have been thus identified for APN: AdipoR1, AdipoR2, and T-cadherin.

AdipoR1 and AdipoR2 receptors are ubiquitously expressed and largely on female reproductive tissues, including ovary, placenta, endometrium, and oviduct [29]. It has been shown that APN inhibits LH and GNRH release, indicating its possible role in modulating the central reproductive endocrine axis [30].

Circulating APN levels decrease with obesity and increase with weight loss and major effects of APN are devoted to increase the insulin sensitivity by stimulating glucose uptake in liver and muscles, while decreasing hepatic gluconeogenesis, and promoting the fatty acid β-oxidation in the skeletal muscle. Consequently, APN reduces triglyceride (TG) accumulation and enhances the insulin sensitivity [31].

Reduced expression of AdipoR1 and AdipoR2 has been also observed in endometria of women with recurrent implantation failure compared with fertile women, suggesting an important role of APN signaling in uterine receptivity and its possible contribution to implantation failures and pregnancy loss in women with maternal metabolic conditions such as obesity and PCOS [32].

- ***Resistin*** *-* Resistin is a small cysteine-rich 94-amino acid polypeptide. This adipokine is considered a potential link between obesity and T2DM as result of its inhibitory effect on adipocyte differentiation and its association with IR. Resistin is mainly secreted by peripheral blood mononuclear cells including macrophages and by stromal cells in adipose tissue, but its mRNA has been also found in hypothalamus-pituitary axis [33].

The resistin gene polymorphism is associated with BMI in women with PCOS, suggesting that it might be related to adiposity in PCOS. A randomized placebo-controlled study recently showed that treatment with the insulin sensitizer rosiglitazone significantly reduces the serum resistin levels in overweight women with PCOS, thus implying the contribution of this adipokine to the insulin sensitivity improvement during treatment [34]. In summary, resistin seems to be an important adipokine that is involved in obesity, IR, PCOS and endocrine dysfunction.

- ***Visfatin*** *-* Visfatin is a protein expressed by a variety of tissues and cell types, including adipocytes, lymphocytes, bone marrow, liver, muscle, trophoblast, and fetal membranes.

In vitro studies have demonstrated that visfatin stimulates the glucose uptake in both adipocytes and muscle cells while suppressing the release of glucose by hepatocytes. A recent meta-analysis revealed that plasma visfatin is significantly increased in both overweight and obese subjects, or in patients with T2DM and metabolic syndrome [35]. It has been also reported that the gene expression and the circulating levels of visfatin are increased in women with PCOS as compared with age and BMI matched controls. A positive correlation, however, has been found between plasma visfatin concentration and fasting insulin in a homeostasis model assessment (HOMA)-IR [36].

- ***Omentin*** *-* Omentin-1, also named intelectin-1, a 313-amino acid peptide, is an anti-inflammatory adipokine preferentially expressed in stromal vascular cells of visceral adipose tissue. It is suggested that this substance makes an important contribution to the physiological difference between visceral and subcutaneous adipose tissue. It is also abundant in vasculature, small intestine, colon, thymus and heart.

Omentin-1 is the major circulating form but other homologues are known. Genes of both omentin-1 and omentin-2 are located as adjacent to each other at 1q22-q23, exactly in the region linked to type 2 diabetes mellitus. Both omentin homologues as circulating forms correlate with expression in visceral fat tissue [37]. Omentin-1 increases the insulin signal transduction as well as the insulin-stimulated glucose transport in human adipocytes though with no effect on basal glucose uptake, and contributes to the regulation of lipid metabolism. Its role, however, in the reproduction’s physiology is unclear.

#### Dysendocrine effects of obesity: hyper- and hypo-androgenism

- ***Obesity, hyperandrogenism and anovulation*** - Obesity, body fat location as upper versus lower body disposition, and muscle mass have important effects on the reproductive function and fertility. The majority of obese women have normal ovulatory menstrual cycles, remain fertile and have no apparent hyperandrogenism thus suggesting that obesity per se is not the only factor involved in the genesis of hyperandrogenism and ovulatory dysfunction. However, elevated androgen levels in obese women appear to be a common finding in the presence of amenorrhea. The mechanism by which the excess adipose tissue can be associated with hyperandrogenism remains controversial although the hyperinsulinemia plays a primary role in influencing obesity in hyperandrogenism.

**Fig. 2:**
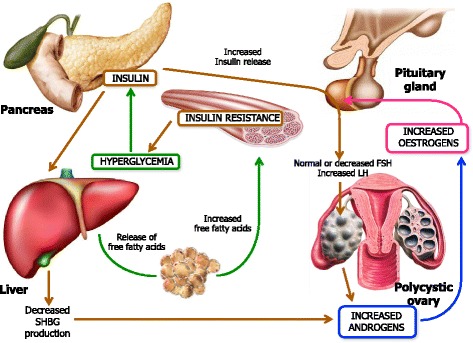
Pathophysioly of insuline resistance. This figure illustrates the complex interactions underlying the pathophysiology of PCOS. Insulin resistance and the resulting hyperinsulinemia are responsible for the majority of the changes found in PCOS. Insulin resistance in PCOS does not occur in all tissues, but rather appears to be tissue-specific. Skeletal-muscle and adipose tissue become insulin resistance resulting in decrease glucose uptake and increased lipolysis, respectively, whereas the ovary, adrenal and liver remain insulin sensitive. In PCOS, hyperinsulinemia occurs as a compensatory response to insulin resistance. This resulting hyperinsulinemia has a stimulatory effect on the ovaries and adrenal glands that leads to enhanced androgen production by these organs. More specifically, excess insulin enhances androgen production in ovarian theca cells in response to luteinizing hormone (LH) stimulation, resulting in follicular arrest and anovulation. In contrast hyperinsulinemia acts to suppress hepatic production of sex hormone-binding globulin (SHBG), the primary binding protein for testosterone in the serum. Therefore, insuline resistance whith compensatory hyperinsulinemia results in hyperandrogenemia

Hyperinsulinemia and insulin resistance are the underlying causes that lead to obesity, accompanied by hyperandrogenism and alterations in steroidogenesis, in those patients with stromal hyperplasia of the ovaries. Both hyperinsulinemia and hyperandrogenism affect the ovarian function in both obese and non-obese women although the mechanism by which how hyperandrogenism and/or hyperinsulinemia inhibit the regular ovulation has not been fully understood. It has been experimentally shown that insulin has specific actions on steroidogenesis through its own receptor since it stimulates the theca cells to produce androgens and exert a growth stimulatory effect on stromal cells, thus priming the production of extradiol [38] Fig. 2. Another proposed mechanism of hyperandrogenism induced by hyperinsulinemia, which can be found in the PCOS patient obese or not, occurs through the insulin-like growth factor-I (IGF-I) which is secreted by human ovarian tissue while its receptors are also located in ovaries.

**Fig. 3:**
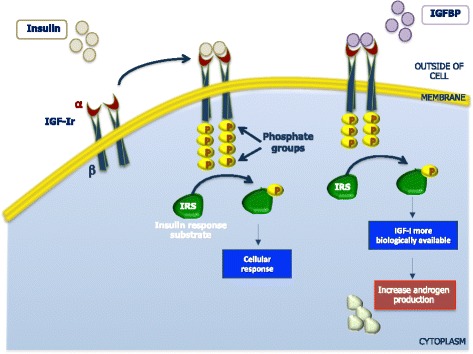
Insulin receptor tyrosine kinases. The α subunit binds insulin and the β transmits a signal from bound insulin to the cytoplasm. The insulin signal activates the receptor’s protein kinases domain in the cytoplasm. Proteine kinases from the receptor phosphorylate insuline-response substrates triggering other chemical responses inside the cell. When IGFBPs binds and therefore activates the IGF-Ir, the hepatic synthesis of IGFBP-I is decreased, making IGF-I more biologically available, thus enhancing androgen production by theca interstitial and stromal cells

Insulin can bind IGF-I receptors (IGF-IR) as well as its own receptors and activates the tyrosine kinase of the β-subunit resulting in triggering the intracellular events that potentiate those normally mediated by IGF-I. Therefore, the insulin-like growth factor binding proteins (IGFBP) include a group of secreted proteins which bind to IGF-I and IGF-II with high affinity and modulate the biological actions of IGF [38]. When the IGFBPs bind and activate the IGF-IR, the hepatic synthesis of IGFBP-I is decreased thus making IGF-I more biologically available with the final effect of enhancing the androgen production by theca interstitial and stromal cells Fig. 3.

Willis et al., showed that insulin also reinforces the activity of the LH on granulosa cells by exerting two distinct effects on the preovulatory follicle, namely the steroidogenesis activation and the inhibition of mitosis thus restraining the terminal differentiation of those cells [39]. As a result of the enhanced steroidogenesis due to insulin and its interaction with LH, the unfavorable milieu produces the blockage of the follicle growth. Thus, the premature luteinization and the consequent follicular arrest result in menstrual cycle disorders and oligo-anovulation which appears strictly related to the obesity [40]. The increased estrogen production, due to peripheral conversion, impairs the function of HPG axis and renders both estrogen excess and hyperandrogenism as major causes of anovulation in these patients.

A nature-experiment of the association obesity/infertility is related to the Laron syndrome (LS), a rare autosomal recessively inherited disease described in consanguineous families originating from Mediterranean, middle-east or south Asian regions [41]. This syndrome is characterized by a primary GH insensitivity or resistance caused by deletions or mutations in the GH receptor gene, which leads to absence of GH activity and congenital IGF-I deficiency [42]. However, one of the intriguing characteristics of LS, in addition to dwarfism, is the chronic obesity which occurs in early childhood and does not decrease with long-term IGF-I treatment. In parallel with a remarkable increase of both subcutaneous and visceral fat, the majority of patients with LS progressively develop signs of metabolic syndrome as hyperlipidemia and non-alcoholic fatty liver disease. Based on this deeply altered metabolism of lipids, the women with LS are usually subfertile and require a treatment to induce ovulation for the chronic defects in their reproductive function [43]. Therefore, given the tight interconnection between energy metabolism and reproduction, the impact that obesity induces on fertility in these women can be considered an aggravating factor in this clinical condition.

- ***Obesity, menstrual cycle and fertility in hypoandrogenic women*** - Whether most of the studies on menstrual cycle and fertility in obesity have been completed in hyperandrogenic women, very few studies examined women without hyperandrogenism and/or with normal menstrual cycle.

One cross-sectional study performed in more than 250 overweight and obese non-hyper-androgenic women partially claiming problems of infertility, showed that 64% of these women were eumenorrheic with normal menstrual cycles, whereas in the original population 21% of them had oligomenorrhea and only 14% were hyper or polimenorrheic, thus proving that oligomenorrhea is the most frequent alteration of menstrual cycle in apparently fertile obese women [44].

The same study showed that abdominal fat accumulation measured by waist circumference, is the most predictive parameter of oligomenorrhea in apparently fertile women, independently from hyperinsulinemia, insulin resistance or other parameters. However, obese non-hyperandrogenic women, even with normal menstrual cycles and apparent normal fertility, have lower circulating levels of gonadotropins, estradiol and inhibin during the follicular phase, thus suggesting an inhibitory effect of obesity per se on the production of these hormones [45]. By contrast, the number of ovary follicles does not seem to be influenced by insulin and body mass in these patients 46].

Lastly, free testosterone (FT) levels are usually higher in obese premenopausal women without hyrsutism and normal/lower circulating levels of other androgens including dehidro-epiandrosterone (DHEA), DHEA-sulphate, androstenedione and 17-OH-progesterone [46], raising the possibility that increased concentrations of FT may have a negative effect on fertility. DHEA is a hormone intermediary in the testosterone- and estrogen-pathway. Its plasma levels are significantly lower in morbid obesity [47], and are inversely correlated with total body fat. These relationships might be the expression of a lower production of DHEA by adrenal glands and/or of higher DHEA uptake in adipose tissue, as well as of a lipomobilizing effect of DHEA. On the other hand, this hormone stimulates resting metabolic rate (RMR) and lipid oxidation at the level of visceral adipose tissue in particular, and enhances the glucose disposal by increasing the expression of GLUT-1 and GLUT-4 on fat cell plasma membrane [48]. For these reasons several IVF units administer DHEA to improve the endocrine environment and the influences on the oocytes.

Therefore, on the basis of these information, it is evident that the decrease of DHEA levels may furtherly enhance the fat accumulation, thus generating a vicious circle in obesity and contributing to higher insulin concentrations and lower fertility in obese women.

#### Obesity effects on reproduction apparatus

Obesity may affect the woman fertility by a number of events as impairment of ovarian follicles development, qualitative and quantitative defects of oocyte maturation, altered fertilization, disrupted meiosis and mitochondrial dynamics derangements leading to abnormal embryo preimplantation [49]. The excess of free fatty acids may exert a toxic effect in reproductive tissues producing a persistent cell damage and a chronic low-grade inflammatory state. To this regard, several mechanisms have been postulated to explain why obesity and/or the overweight affect the quality of female gametes and several hypotheses are related to plasmatic levels of reproductive hormones and their metabolism.

In this contest, Machtinger et al., explored the oocytes failed to be fertilized in IVF cycles of morbidly obese women, and described disarrayed meiotic spindles with misaligned metaphase chromosomes [50]. Independently from aneuploidy, obesity also appears to alter the mitochondrial function in the oocyte. One potential mechanism for oocyte organelle damage in obesity is the lipotoxicity. In fact, the excess fatty acids obtained from the diet can be stored as triglycerides in adipocytes although they are apparently unable to induce the cellular damage in this storage compartment. However, when this capacity is overwhelmed with continued dietary excess, fatty acids accumulate in other tissues and exert their toxic effects known as lipotoxicity [51].

Furthermore, obese women have higher levels of circulating free fatty acids, which damage non adipose cells by increasing reactive oxygen species (ROS) that, in turn, induce mitochondrial and ER stress resulting in apoptosis of multiple cell types including oocytes [52]. This effect is related to the chronic low grade inflammatory state related to obesity which is proven by the increased circulating levels of C-reactive protein (CRP), as well as lactate and triglyceride concentrations in follicular fluid, and by the enhanced expression of pro-inflammatory (CXCL2) and oxidative stress related (DUSP) genes [53].

The preimplantation embryo is also affected by the endometrial obese environment. Comparison of human IVF cycles with autologous oocytes show that obese women are more likely to create poor quality embryos [54] as effect of the lipotoxicity of embryonic cells in a similar fashion as described for the oocyte. However, there are conflicting data as to whether obesity has a significant effect on the endometrium. Although many factors contribute to restrain reproductive outcomes in obese women, Rhee JS et al., in their study emphasize the importance of decidualization defects in obese women which would alter the endometrial receptivity resulting in poor implantation [55].

### The ‘at risk’ fertility in obese women

Although in a high percentage of obese women the pregnancy occurs with normal accomplishment, several risks in their conception plans need to be considered and are briefly discussed.

#### Impact on egg viability and quality

The constitutive ovarian reserve in fertile women is not unlimited and the number of maturing oocytes usually declines with aging. For this reason, the impact of maternal environment and obesity on the differentiation of these cells is particularly consequential and negative environmental exposures may affect the developmental competence of the oocyte, defined as the ability of mature oocytes to be fertilized and support the embryo development [56]. Thus, based on the systemic inflammatory state associated to the obesity, the oocyte maturation is variably affected by the altered balance of driver hormones as SHBG with other soluble factors including insulin, glucose, lactate, triglycerides, and C reactive protein [53].

High BMI values also alter the concentration of certain adipokines. In rodent models, high concentrations of leptin impair the follicle development, ovulation, and oocyte maturation [57], whereas leptin deficient mice (ob/ob) are the animal experimental model to investigate the effects of obesity, and female ob/ob mice develop low numbers of antral follicules resulting in impaired folliculogenesis, reduced ovulation rate, and accelerated apoptosis of granulosa cells [58].

Furthermore, elevated levels of leptin associated with high BMI also impairs the steroidogenesis. The mechanisms by which leptin regulates the steroidogenesis in human granulosa cells have been investigated by Lin Q. et al., in experimental studies completed in 40 granulosa cell lines obtained from explanted human granulosa samples during in vitro fertilization program [59]. They show that human granulosa cells after exposure to high dosage of recombinant human leptin, inhibit 8-bromo cAMP-stimulated progesterone production in a dose-dependent manner with downregulation of StAR mRNA levels, a steroidogenic regulatory protein, produced for the progesterone synthesis. Therefore, they suggest that leptin interferes with the secretion of gonadotropin-stimulated proge-sterone in these cells and with induction of StAR protein by cAMP which, thus, is significantly reduced. They also proved that both cAMP-regulated steroidogenic enzymes and progesterone production could be inhibited by leptin via the MAPK pathway. These results confirm that leptin acts through its receptor to initiate the MAPK pathway and downregulates the cAMP-induced steroidogenesis in human granulosa cells [59].

Leptin receptors are structurally similar to the class I cytokine receptor family [53]. In humans, the leptin receptor (OB-R) is produced in several alternatively spliced forms, designated OB-Ra, OB-Rb, OB-Rc, OB-Re [60]. Each of these isoforms shows an extracellular domain and a transmembrane domain in common, with a variable intracellular domain typical for each isoform. Based on the variable intracellular domain, these isoforms are classified as short (OB-Ra), long (OB-Rb) and secreted (OB-Re) leptin receptor. Other than the classical JAK/STAT signalling pathways, leptin may act through OB-Ra or OB-Rb to trigger the MAPK cascade in two different ways, namely via tyrosine phosphorylation of JAK2 receptor-associated activation, or independently from the receptor phosphorylation [61]. Thus, the stimulation of granulosa cells by leptin was used to investigate the activation of MAPK pathway suggesting that MAPK cascades are involved in leptin mediated inhibition of steroidogenesis in granulosa cells by the phosphorylation of ERK, p38 and JNK.

Adiponectin is primarily secreted by adipocytes, but its serum levels drop down in obesity and in insulin resistance while increasing with weight loss [62]. It has been demonstrated that in obese women where circulating levels are lower, there would just reflect the insulin sensitivity and that this effect may negatively influence the control of ovulation since adiponectin is variably detectable in ovaries, follicular fluid, oocyte, corpus luteum, theca cells, while is weakly expressed by granulosa cells [63]. However, the direct effect of adiponectin in ovarian function remains unclear. In animal models, the protein has been described to influence both folliculogenesis and the follicular remodelling as well as to modulate the sex steroid secretion via activation of its own receptors R1 and R2, and by modulating the insulin/IGF system [64].

Ledoux and co-workers examined the effects of recombinant porcine adiponectin on porcine ovarian granulosa cells in vitro and demonstrated that the protein at physiologically levels equal to 10–25 μg/ml, provokes expression of genes committed to the periovulatory remodelling of the ovarian follicle over a time frame of 6-24 h. These include cyclooxygenase-2, prostaglandin E synthase, and vascular endothelial growth factor. Therefore, adiponectin modulates steroid synthetic protein gene expression by increasing the steroidogenic acute regulatory protein transcript and, concurrently, by reducing the cytochrome P450 aromatase. Finally, these studies demonstrated that the MAPK pathway, via phosphorylation of ERK1/2, is involved in mediation of the adiponectin signal in ovarian granulosa cells, rather than the protein kinase A, or the classic adiponectin transducer AMP-activated protein kinase. These results suggested that since adiponectin synthesis is reduced in obesity, this defect may play a substantial role in obesity-related ovarian dysfunction [64].

Other adipokines such as interluekin-6 (IL6), plasminogen activator inhibitor (PAI) type-1, or TNF family members may affect oocyte competence or maturation through alterations in steroidogenesis and interaction with other metabolic hormones [65]. While the mechanisms by which adipokines impact the oocyte quality have not been elucidated yet, their altered concentrations due to obesity represent the major potential factor by which obesity may negatively impact on the oocyte health thus affecting the fertility in women.

#### Obesity and miscarriage rate

The association between obesity and miscarriage has been assessed in a number of studies in both natural and assisted conceptions in which the miscarriage risk was as high as up to approximately 40% in obese women with respect to less than 15% in females with a normal BMI. However, despite these findings, there is no consensus about the causative mechanisms in obese women [66].

A major pathogenic factor could be related to the impaired stromal decidualization in obese women which is responsible of placental abnormalities, stillbirth and preeclampsia [67], although the most frequent cause of first trimester miscarriages usually related to embryo chromosomal pathologies, does not seem to be increased in overweight women [68].

#### AMH in obese patients

Anti-Mullerian hormone (AMH) is a product of the granulosa cells of small antral and pre-antral follicles, and clinically, it may be reflective of the prediction of ovarian reserve in women undergoing fertility evaluation and treatment [69]. For this reason, is important to evaluate the change in the levels of AMH, as a fertility parameter in obese women with or without PCOS, submitted to aerobic exercise with the aim of losing weight. The slimming via exercise or diet is considered one of the most important targets in lifestyle modification programmes capable to induce an improvement in reproductive function among obese women with PCOS [70]. Exercise interventions of moderate activity are one of the most important lifestyle modifications that positively influence on fertility and assisted reproductive technology outcomes [71].

Einas AE and coworkers, have studied the possible correlation with adiposity, clinical and hormonal parameters in PCOS and normo-ovulatory women, conducting a one-year study among obese women with or without polycystic ovary syndrome. All patients were classified into three age-matched groups; group A: controls, group B: PCOS patients and group C: obese women. The AMH, was measured at baseline and following 12 weeks of supervised aerobic exercise. They conclude that the change in AMH levels correlated significantly with physical activity, therefore the authors claim that a moderate aerobic training for 12 weeks had a positive significant effect on reproductive functions via modulating adiposity, AMH and fertility in obese women with or without PCOS [72].

#### Influence of obesity on fertility treatments

Overweight and obese women have lower outcomes following fertility treatments than normal population. They poorly respond to induction of ovulation, require higher doses of gonadotropins and longer treatment courses for follicular development and ovulatory cycles. In addition, the oocyte yield is lower in obese women resulting in a higher rate of cycle cancellation [73]. Ovarian stimulation for assisted reproduction produces fewer follicles leading to the harvest of fewer oocytes. Thus, the fertilization rates are scarce and the embryo quality is impaired in younger obese undergoing fertility treatments who definitely show low pregnancy rate with increased risk of early pregnancy loss.

Glueck CJ et al., report in their study that insulin sensitising-lowering agents ameliorate the reproductive, and metabolic disorders, typical of the obese patient [74]. The action of metformin is widely discussed in the literature, which is considered as the first-line medication for the treatment of type 2 diabetes. Women with PCOS receiving metformin improved pregnancy outcomes, and this has been attributed to its ability to reduce the insulin resistance as well as hyperinsulinaemia and the inhibitor of the hypofibrinolytic plasminogen activator resulting in improvement of oocyte quality and folliculogenesis for amelioration of PCOS.

The major effects of metformin include its property to decrease the liver glucose production by suppressing hepatic gluconeogenesis as well as the increase of insulin sensitivity and peripheral glucose uptake [75]_._ The molecular mechanism of metformin is incompletely understood. Multiple potential mechanisms of action have been proposed, including; inhibition of the mitochondrial respiratory chain (complex I), activation of AMP-activated protein kinase (AMPK), inhibition of glucagon induced elevation of cyclic adenosine monophosphate (cAMP) with reduced activation of protein kinase A (PKA), inhibition of mitochondrial glycerophosphate dehydrogenase, and an effect on gut microbiota [76]. All these activities may have contributed to improve the oocyte maturation in PCOS and may exert a definitive favorable effect on infertility associated to inefficient oocyte differentiation and maturation.

### Benefits from weight loss

Weight loss has been shown to improve reproductive outcomes by ameliorating fertility, as well as by regularizing menstrual cycles and increasing the chance of spontaneous ovulation and conception in anovulatory overweight and obese women.

Available data suggest that the weight loss equal to 5%–10% of the body weight may definitely improve the fertility rate [77], whereas other studies prove that 5% of weight loss results in significant improvement of endocrine parameters, such as decrease of free testosterone, and LH and insulin levels, with the improvement of ovulation frequency [78].

Sim and collaborators investigated the effects of weight loss in overweight and/or obese women undergoing assisted reproductive procedures on their subsequent pregnancy outcome. In their observation, the weight loss achieved by diet and lifestyle change resulted in significantly increased pregnancy rates and/or live birth in overweight and/or obese women undergoing assisted reproductive technology (ART) in 8 of the 11 studies reviewed. In addition, regularization of the menstrual pattern, decrease in cancellation rates, increase in number of embryos available for transfer, reduction in the number of ART cycles required to achieve pregnancy, and a significant decrease in miscarriage rates were reported. Interestingly, an increase of the number of natural conceptions in five of the six studies was also reported [79].

However, the decision to postpone the treatment of fertility to allow weight loss often results in a further increase in maternal age in women who are not very young. A recent study demonstrated that the effect of BMI on IVF success was strongly influenced by age. In fact, the effect of BMI reduction alone is apparently reduced with increasing age, and in women aged 36 yrs. or older, the weight loss may sometimes result not functional in favouring the woman fertility [80].

## Conclusions

Overweight and obese women need longer time to conceive and undoubtedly are at higher risk of infertility. Higher BMI is also associated with adverse pregnancy outcomes, such as gestational diabetes and hypertension and women undergoing undergoing in vitro fertilization may experience negative outcomes at higher rate than normal weight females. However, as early symptoms of dysfunctional oocyte maturation and hormone derangements, oligomenorrhea and alterations of menstrual cycles should primarily alert overweight and obese women on their potentially defective fertility. The impact of obesity on reproductive function, especially associated with ovulatory disorders, is mainly due to neuroendocrine mechanisms, which interfere with ovarian functions, and are able to affect the ovulation rate and the endometrial receptivity. Obese women, even with normal menstrual cycles and apparently normal fertility, have lower circulating levels of gonadotropins, estradiol and inhibin in the follicular phase, suggesting an inhibitory effect of the obesity condition per se on the production of these hormones.

Since the obesity is pathogenically associated to inflammation, all mechanisms enrolled in oocyte differentiation and maturation including hormones, proteins and soluble factors released by adipocytes are deregulated and affected in their physiology. The direct effect of adipose tissue in lowering the fertility potential in women is thus based on the derangement of major molecular mechanisms that regulate the normal biological activity of cell components of the woman reproductive organs also controlled through the hypothalamus hypophysis ovaries axis.

In conclusion, in terms of human and social impact of obesity in women during the reproductive phase, this disease does not affect only the cardiovascular system and the skeleton health for higher incidence of osteoporosis and dramatically restrains the physical activity in addition to the unfavourable aesthetic impact in women, but also provides suffering in relation to the lower probabilities to conceive and normal pregnancies as in non-obese females.

## Abbreviations

IL1β: Interleukin 1β
TNFα: Tumor necrosis factor α
AMH: Anti-Mullerian hormone
AMPK: AMP-activated preotein kinas
APN: Adiponectin
APN-LMW: Adiponectin at low molecular weight
APN-MMW: at medium molecular weight
APN-HMW: at high molecular weight
ART: Assisted reproductive technology
BMI: Body mass index
cAMP: Cyclic adenosine monophosphate
DHEA: Dehidro-epiandrosterone
FT: Free testosterone
GH: Growth hormone
HOMA: Homeostasis model assessment
HPO: Hypothalamic-pituitary-ovarian
IGFBP: Insulin-like growth factor binding proteins
IGF-I: Insulin-like growth factor-I
IGF-IR: IGF-I receptors
IL6: Inter-leukin 6
IL6: Interluekin-6
IR: Insulin resistance
LCN2: Lipocalin-2 (LCN2)
LEPR: Leptin receptor
LS: Laron syndrome
OSHA: Occupational Safety and Health Administration
PAI: Plasminogen activator inhibitor
PCOS: Polycystic ovary syndrome
PKA: Protein kinase A
RBP4: Protein-4
RMR: Resting metabolic rate
ROS: Reactive oxygen species
SHBG: Sex hormone binding globulin
SHBG: Sex hormone-binding globulin
T2DM: Type 2 diabetes mellitus
TG: Triglyceride
